# Effects of a three-year maize-soybean rotation on soil bacterial diversity and fumonisin-producing *Fusarium* spp. inoculum potential

**DOI:** 10.1007/s11274-026-05358-2

**Published:** 2026-10-20

**Authors:** Kize Alves Almeida, Rafaela Araújo Guimarães, Victor Biazzotto Correia Porto, Luiz Flávio Machado Góes, Fatima Maria Souza de Moreira, John Quensen, James Tiedje, Flávio Henrique Vasconcelos de Medeiros

**Affiliations:** 1 Department of Phytopathology, Laboratory of Biological Control of Plant Diseases, Universidade Federal de Lavras (UFLA), Lavras, 37200-000 MG Brazil; 2 Department of Soil Science, Sector of Biology, Microbiology and Biological Processes, Universidade Federal de Lavras (UFLA), Lavras, 37200-000 MG Brazil; 3 Department of Plant, Soil and Microbial Sciences, Center for Microbial Ecology, Michigan State University, East Lansing, MI 48824 USA; 4 Department of Phytopathology, Universidade Federal de Lavras (UFLA), Lavras, 37200-000 MG Brazil

**Keywords:** Crop rotation, Soil microbiome, Fusarium stalk rot, fum1, Rhizosphere, No-tillage

## Abstract

Maize-soybean succession dominates grain production in tropical Brazil, and rotation is often recommended to improve soil health. However, few field studies have followed both soil bacterial diversity and fumonisin-producing pathogens over several seasons in tropical no-till systems. We compared continuous maize, continuous soybean and maize–soybean rotation for three seasons (2016–2017 to 2018–2019). In a fourth season (2019–2020), maize was grown in all plots to test legacy effects. Bacterial communities were profiled by 16 S rRNA (V3-V4) amplicon sequencing. The fum1 gene of fumonisin-producing *Fusarium* spp. was quantified by qPCR in soil and in maize stalks left on the soil surface. In the first three seasons, rotation did not detectably increase bacterial diversity, although statistical power was low (*n* = 4 plots; post-hoc power 12–61%). Community composition differed among seasons and was associated with antecedent precipitation as Actinobacteria were most abundant in the driest season. In the fourth season, a continuous-soybean history was associated with higher bacterial richness and Shannon diversity in the maize rhizosphere at tasselling than a rotation history, whereas bulk-soil diversity did not differ among land uses. A rotation history was associated with lower fum1 abundance in bulk soil. In contrast, continuous maize was associated with lower fum1 in stalks (third season), less stalk rot and fewer infected grains. Soil and crop residues therefore responded differently to rotation. Given the single site and limited plot-level replication, these findings should be regarded as hypothesis-generating.

**Supplementary Information:**

The online version contains supplementary material available at https://doi.org/10.1007/s11274-026-05358-2.

## Introduction

Crop succession and no-till systems are widely used as strategies to improve soil quality, control weeds and pests, and increase plant productivity (Tiemann et al. 2015; Pittelkow et al. 2015; Du et al. 2023). These practices have been found to increase belowground diversity, thereby increasing resilience and improving plant performance under extreme weather conditions such as drought (de Vries et al. 2020; Sokol et al. 2022). They can also promote soil suppressiveness by altering the composition and abundance of key microorganisms (Peralta et al. 2018; Zhang et al. 2023).

Soybean and maize are the most important crops produced in Brazil (Junior and Centelhas 2019; CONAB 2025), often cultivated in succession or rotation due to their high profitability and suitability to regional climate conditions. However, cropland expansion for these and other commodities has been rapidly increasing, particularly in biomes such as the Amazon, Caatinga, and Cerrado (Zalles et al. 2019; MapBiomas 2023, 2024). According to the Companhia Nacional do Abastecimento-Brazil (CONAB), the 2018–2019 season recorded increased production and cultivated area for soybean, while maize production showed a slight decline. More recently, the 2024–2025 season set a new national grain production record of 345.2 million tonnes, driven largely by record soybean (169.7 million tonnes) and increased maize output, underscoring that the expansion trend described above has continued into the current decade (CONAB 2025).

Crop succession and no-tillage are widely recognized strategies to reduce soil exhaustion, enhance nutrient cycling, and mitigate pest pressures (de Faccio Carvalho et al. 2010; Lal 2020). However, rotational systems that include no-till practices may inadvertently increase pathogen inoculum due to the retention of plant residues on the soil surface (Munkvold 2003; McDonald and Stukenbrock 2016). Nevertheless, research suggests that crop rotation enhances microbial diversity and may promote suppressiveness against soilborne pathogens (Garbeva et al. 2004; Vukicevich et al. 2016; Borrelli et al. 2021). Soil microorganisms are known to protect plants from diseases (Krupinsky et al. 2002), and soil microbiome composition is influenced by variables such as pH, moisture, texture, organic carbon, and management practices (Peralta et al. 2018; Davey et al. 2019; Banerjee et al. 2023). Additionally, studies indicate that increases in aboveground diversity correspond to enhanced belowground microbial diversity, which is linked to soil and plant health (Nielsen et al. 2015; Bardgett and van der Putten 2024).

*Fusarium verticillioides* (Sacc.) Nirenberg is a major economic threat to maize production and grain quality. The fungus survives in soil and crop residues, elevating inoculum potential in subsequent seasons. It infects maize through seeds, wounds, or natural openings, causing ear rot and resulting in characteristic starburst kernel patterns (Pedrozo and Little 2017; Blacutt et al. 2018; Munkvold 2021).

Despite this evidence, important gaps remain. Most studies on crop rotation, soil microbial diversity and pathogen suppression are short-term (a single season or a greenhouse assay) or come from temperate systems (Benitez et al. 2017, 2021). They usually report either community shifts or pathogen suppression, rarely both in the same design. It is also unclear whether rotation-induced changes in bacterial diversity persist into a following monoculture season. Whether such legacy effects reduce a mycotoxin-producing pathogen in soil and crop residues is even less known, particularly in tropical no-till maize-soybean systems (Khan et al. 2025; Yin et al. 2026). We addressed this gap with a four-season field study that combined 16 S rRNA profiling of bulk soil and rhizosphere, qPCR quantification of fumonisin-producing *Fusarium* spp. (fum1) in soil and maize stalks, and assessment of stalk rot and grain health.

This study aimed to evaluate the impact of a three-year maize-soybean crop rotation on soil microbial diversity and fumonisin-producing *Fusarium* spp. (fum1) inoculum potential in both soil and maize stalks. A microbiome-guided approach (16 S rRNA sequencing) was used to assess bacterial soil diversity under rotational and monocropping systems. Additionally, specific primers targeting the fumonisin gene (fum1) were applied to quantify fumonisin-producing *Fusarium* spp. inoculum in total DNA samples extracted from soil and stalks. This knowledge will contribute to better understanding and management of *F. verticillioides* in tropical agricultural systems.

## Materials and methods

### Site description and experimental design

Field experiments were conducted at the Centro de Desenvolvimento Científico e Tecnológico em Agropecuária, at the CDCTA – Centro de Desenvolvimento Científico e Tecnológico em Agropecuária (Universidade Federal de Lavras, Minas Gerais, Brazil). The predominant soil type at the site is classified as Cambissolo Háplico (Brazilian Soil Classification System), corresponding to an Inceptisol under USDA Soil Taxonomy. The regional climate is classified as Cwa (humid subtropical, with dry winters and hot summers) according to the Köppen-Geiger system (Alvares et al., 2013). Across the four growing seasons, total precipitation and mean air temperature (recorded at the local meteorological station) were: season 1 (2016–2017), 557.1 mm, 23.2 °C; season 2 (2017–2018), 732.7 mm, 22.8 °C; season 3 (2018–2019), 1308.4 mm, 22.7 °C; and season 4 (2019–2020), 1076.9 mm (partial record, 117 of 157 days), 23.2 °C (Supplementary Table S1).

Although its seasonal total was higher than that of season 1, season 2 had a markedly dry period in the 14 days immediately preceding harvest sampling (0–3 mm). Soil chemical properties, determined for all 12 plots at the end of the experiment (accredited laboratory, Lavras-MG; sampled June 2020), were (mean ± SD, *n* = 12): pH (H₂O) 5.72 ± 0.32; available P (Mehlich-1) 14.9 ± 1.9 mg/dm³; available K 354.4 ± 30.7 mg/dm³; exchangeable Ca 4.95 ± 0.47, Mg 1.32 ± 0.30, and Al 0.05 ± 0.05 cmolc/dm³; H + Al 2.88 ± 0.66 cmolc/dm³; organic matter 4.33 ± 0.42%; sum of exchangeable bases (SB) 7.18 ± 0.77 cmolc/dm³; cation exchange capacity at pH 7.0 (CTC/T) 10.06 ± 0.87 cmolc/dm³; and base saturation (V) 71.5 ± 5.6%. These values were comparable across the three land-use treatments (Supplementary Table S2), Because these samples were collected after the four seasons, they do not represent pre-experiment baseline conditions; they indicate only that land-use history did not produce marked differences in soil fertility that could confound treatment comparisons. The chosen area had a history of 10 consecutive years of maize cultivation and the incidence of ear and stalk rots; stalk rots were confirmed by Pinto et al. (2022) based on morphological characterization and disease symptom development.

The experimental assay was conducted over four years (2016–2017 to 2019–2020), with the first three years marked by soil cultivation history, including maize monoculture, soybean monoculture, and maize-soybean succession, all under a no-tillage system. In the fourth year, the entire area was dedicated to maize monoculture. The experiment was designed with four complete randomized blocks within 15 m² plots, with five rows 0.6 m apart. Maize hybrid DKB290 VTPRO3 (Dekalb Brazil) and soybean NS5700 IPRO (Nidera Seeds) were planted at densities of 65,000 and 300,000 plants ha⁻¹, respectively, in all seasons. Soil and stalk samples were collected at harvest time (grain moisture content of approximately 22% for maize and 18% for soybean).

Bulk soil samples were collected at harvest in the first three seasons (2016–2017 to 2018–2019); in the fourth season (2019–2020), both bulk soil and rhizosphere samples were collected at two time points, blooming (maize tassel stage, VT) and harvest. In the last season, maize was planted across the entire experimental area to compare the impact of three-year land uses on soil diversity and the fum1 gene. For the first three years, ten randomized samples per plot were combined into a composite sample, totaling 36 samples. In the fourth season, 12 sampling points per plot (bulk and rhizosphere) were combined into three composite samples per plot, yielding 36 bulk and 36 rhizosphere samples per time point (4 blocks × 3 treatments × 3 composite samples), totaling 72 bulk and 72 rhizosphere samples when summed across both the blooming and harvest time points. Bulk soil was collected between plant rows, away from the plants, with a soil auger at 0–10 cm depth after the surface crop residues had been removed; each composite sample combined 10 (seasons 1–3) or 4 (season 4) cores taken in a zigzag pattern within the three central rows of each plot.

Rhizosphere samples were collected from maize plants in the same rows. The root system of each plant was carefully excavated with a shovel, loosely attached soil was removed by gently shaking the roots, and the soil tightly adhering to the roots (rhizosphere soil) was then detached by vigorous shaking and brushing with a sterile brush into sterile plastic bags; four plants were combined to form each rhizosphere composite sample. Each bulk-soil core contained approximately 100 g of soil, and all rhizosphere soil recovered from each plant (approximately 10–20 g) was used. Subsamples from the same plot (seasons 1–3) or group of four points (season 4) were placed in a single sterile plastic bag and homogenized by hand mixing, and visible roots, stones and plant debris were removed. Composite samples were transported to the laboratory on the day of collection in insulated boxes with ice packs and, on arrival, were stored at -20 °C until DNA extraction.

In the first three seasons, DNA was extracted from three 0.5 g subsamples of each composite sample, and the extracts were pooled before sequencing. Fourth-season bulk and rhizosphere samples were freeze-dried and sent to the Center for Microbial Ecology, Michigan State University, for DNA extraction. For the fourth season, DNA was extracted within about 3 months (tasselling) and 5 months (harvest) of sampling, whereas samples from the first three seasons remained at -20 °C for at most about three, two and one years, respectively, before DNA extraction. Within each season, all land-use treatments were sampled on the same day and stored and extracted together, so storage time did not differ among treatments (Supplementary Fig. S3).

For chemical analysis, soil samples for each plot were taken at 0–10 cm depth. Ten maize stalk fragments (20–30 cm) were placed in nylon bags on the soil surface of each plot, totaling 12 bags per season. After the plant cycle, stalks were fragmented into 1–2 cm pieces, ground, freeze-dried, and stored at -20 °C until DNA extraction. Precipitation data from a local meteorology station were collected for 14, 10, 7, and 5 days before each soil sampling time point and fitted as environmental vectors onto a principal coordinate analysis (PCoA) of Bray-Curtis dissimilarities calculated from log-transformed ASV counts (envfit, vegan; 999 permutations; Cailliez correction for negative eigenvalues). Only vectors significant at *p* ≤ 0.05 were displayed.

Precipitation data for the fourth season (2019–2020) were only partially recovered from the local station: 10 of the 14 days preceding the blooming sampling point (28 January 2020) were available (total = 62.7 mm), whereas records for the 14 days preceding the harvest sampling point (19 April 2020) were largely unavailable (5 of 14 days), consistent with a gap in local data collection during March-April 2020.

### Soil DNA extraction and sequencing

Genomic DNA was extracted from 0.5 g of soil using the DNeasy PowerSoil Kit (Qiagen Inc.) according to the manufacturer’s instructions and quantified using the dsDNA HS Assay Kit (Invitrogen) on a Qubit 2.0 Fluorometer (Life Technologies, Grand Island, NY, USA). Libraries were prepared according to Illumina’s protocol and analyzed using the V3 kit for the Illumina MiSeq platform (2 × 300 bp). Primers Bakt_341F and Bakt_805R targeting the hypervariable V3-V4 region of the 16 S rRNA gene were used (Klindworth et al. 2013). Soil DNA samples were diluted to a final concentration of 10 ng/µL and sent to Psomagen (Maryland, USA) for sequencing. A total of 180 composite soil DNA samples were processed for 16 S rRNA sequencing across the study (36 bulk soil samples from the first three seasons, plus 72 bulk and 72 rhizosphere samples from the fourth season. Library preparation PCR was performed by the sequencing provider (Psomagen) using the Bakt_341F/Bakt_805R primer pair (V3-V4 region), following the Illumina 16 S Metagenomic Sequencing Library Preparation guide. Amplicon PCR conditions consisted of an initial denaturation at 95 °C for 3 min, followed by 25 cycles of 95 °C for 30 s, 55 °C for 30 s, and 72 °C for 30 s, with a final extension at 72 °C for 5 min. Provider-specific deviations from this standard protocol, if any, were not independently recorded by the authors. All raw data generated in this study were submitted to NCBI (Sequence Read Archive-SRA) under BioProject number PRJNA1122864.

### Quantification of the fumonisin gene (fum1) in soil and maize stalks by qPCR

To quantify the fumonisin gene in soil and maize stalks, we used the primer set Verpro-F (5′-GCCATGCGTCACGGCCAC-3′) and VERTI-R (5′-GGAGTAGACAGGGTATTTGC-3′) (Waalwijk et al. 2008). This primer pair targets the fum1 gene involved in fumonisin biosynthesis and is not species-specific: it detects multiple fumonisin-producing *Fusarium* spp., including *F. verticillioides* and *F. proliferatum*. Accordingly, fum1 quantification data reported throughout this study reflect fumonisin-producing *Fusarium* spp. inoculum potential rather than *F. verticillioides* specifically; species-level identification of *F. verticillioides* in this study was based on morphological assessment. For maize stalks, genomic DNA was extracted from 40 mg of material using the Wizard^®^ Genomic DNA Purification Kit (Promega Corporation) following the manufacturer’s protocol. Quantification was performed in the Rotor Gene 6000 thermocycler (Qiagen) using SYBR Green PCR Mix (Qiagen) in a final volume of 25 µL with 2 µL of DNA template. The amplification program consisted of an initial denaturation at 95 °C for 5 min, followed by 38 cycles of 95 °C for 25 s, annealing at 58 °C for 30 s, and extension at 72 °C for 30 s. Six standard curves were constructed from a log₁₀ serial dilution starting at 0.2 pg/µL of *F. verticillioides* FV425 DNA (Lanza et al. 2014), a fumonisin gene (fum1) producer. For soil samples, the DNA extracted for taxonomic diversity experiments (the “Soil DNA extraction and sequencing” section) was used.

All samples were analyzed in duplicate, including standards and negative controls (water). A melting curve was assessed for each run to confirm primer specificity, and conventional PCR with gel electrophoresis was performed for verification. Samples with undetermined Ct values (no amplification within 38 cycles, i.e., below the detection limit of the assay) were considered below the detection limit and were excluded from the statistical analysis; for this reason, the second-season stalk samples, most of which were undetermined, were not analysed statistically. Amplification efficiency was assessed for each qPCR run performed across all seasons and both compartments (bulk soil and maize stalks), based on standard curve threshold, R², and efficiency values (Rotor-Gene 6000). Across runs, R² averaged 0.99 (range 0.96–0.99) and amplification efficiency averaged 1.04 (SD 0.11; range 0.79–1.22), full per-run values are provided in Supplementary Table S4. Most runs fell within the commonly accepted efficiency range (90–110%), but some fell outside it; because fum1 data are reported as Ct values rather than absolute quantities, fum1 abundance was compared among land-use treatments within each season and compartment only, and not across seasons.

### Quality of maize grains and stalk rot evaluation

The incidence of *F. verticillioides* was assessed using a blotter test following the recommendations of the Manual de Análise Sanitária de Sementes (Brasil 2009). Maize seeds were surface-sterilized with 70% ethanol for 30 s, 2.5% hypochlorite for 3 min, and washed three times with sterilized water to remove superficial contaminants. In total, 400 grains from each plot were distributed over Petri dishes (146 × 21 mm), using 25 grains per plate. A stereoscopic microscope (Zeiss Stemi DV4, magnification 30–80×) was used for pathogen identification. For maize stalk rot evaluation, 10 symptomatic maize plants per plot (7.2 m², three central rows) were sampled at the flowering (blooming) stage. Stalks were evaluated by pressing the first and second internodes and then incubated in a humid chamber for five days in the dark. Recovery of *F. verticillioides* from infected stalks was confirmed by isolation on potato dextrose agar. The number of infected stalks was converted to a percentage and submitted for statistical analysis.

### Bioinformatics and diversity analysis

Primer sequences (Bakt_341F, 5′-CCTACGGGNGGCWGCAG-3′; Bakt_805R, 5′-GACTACHVGGGTATCTAATCC-3′) were removed with Cutadapt v2.8 (Martin 2011) using default parameters (maximum error rate 0.1; minimum overlap 3 nt). Each sequencing run was processed separately. Truncation lengths were chosen with FIGARO (Weinstein et al. 2019) for each run: 244 and 233 nt for forward and reverse reads in run 1, and 250 and 217 nt in run 2. Paired-end reads were merged with USEARCH v11.0.667 (fastq_mergepairs) using default parameters (at most 5 mismatches and at least 90% identity in the overlap, minimum overlap 16 nt, minimum read length 64 nt), yielding merged amplicons of about 430 bp. Merged reads were imported into QIIME2 v2020.2 (Bolyen et al. 2019) and denoised with the DADA2 algorithm (Callahan et al. 2016) as implemented in the q2-dada2 plugin, using default parameters: maximum expected errors (maxEE) of 2.0, truncation quality score (truncQ) of 2, and chimera removal with removeBimeraDenovo in consensus mode; about 79% of reads were retained after denoising. A summary of each sequencing run is given in Supplementary Table S5, and all processing parameters are listed in Supplementary Table S6; raw reads for every sample are available in the NCBI Sequence Read Archive.

The resulting ASVs were classified with the RDP classifier (Wang et al. 2007) (version 2.13, trained on RDP trainset 18), using the default confidence threshold of 0.8. This was the most recent RDP training set available when the sequences were processed in 2020. Phylum names therefore follow the nomenclature of that training set (e.g., Actinobacteria, Proteobacteria, Bacteroidetes); the corresponding validly published names are Actinomycetota, Pseudomonadota and Bacteroidota (Oren and Garrity 2021). Samples were processed across two sequencing runs. The first run included all samples from the first three seasons (2016–2019) together with the blooming-stage rhizosphere and bulk soil samples from the fourth season (2019–2020). The second run comprised only the fourth-season harvest samples (bulk and rhizosphere soil; 3 composite replicates × 4 blocks × 3 cropping treatments = 72 samples).

All statistical comparisons among land-use treatments presented in this study are performed within a single sampling time point and are therefore within-run comparisons; no formal test pools or directly compares samples across the two sequencing runs, and no run-as-covariate correction was applied. Two runs were used because the fourth-season harvest samples were collected on 19 April 2020, after the samples of the first run had been submitted for sequencing. Both runs used the same DNA extraction kit, primers, library protocol, sequencing provider and platform, with a target depth of about 20,000 reads per sample. Alpha-diversity values were consistently higher in run 2 than in run 1 (Supplementary Table S3). For bulk soil sampled at harvest, mean Chao1 was 1,769-2,240 in the third season (run 1) but 4,078 − 4,360 in the fourth season (run 2), whereas run 1 samples were consistent across years (bulk soil at fourth-season tasselling, 1,737-2,112). This doubling between two harvest samplings indicates a substantial run-specific technical effect, most likely related to sequencing depth and processing. For this reason, absolute diversity values and community composition were not compared across runs. A run correction was not applied for two reasons. First, run and sampling time point are completely confounded in the fourth season, so a correction would also remove any real tasselling-harvest difference. Second, each run contained all land-use treatments in balanced numbers (four blocks × three treatments), and every treatment comparison was made within a single run, so run effects cannot bias the treatment comparisons.

Diversity analyses were performed using the vegan: Community Ecology Package v2.5-6 (Oksanen et al. 2019) on R v3.6.1 (https://www.R-project.org/). Alpha and beta diversity were estimated using richness, the Shannon diversity index, and Bray-Curtis dissimilarity. Differences in community structure among land-use treatments were tested by permutational multivariate analysis of variance (PERMANOVA; vegan adonis, 999 permutations), followed by pairwise PERMANOVA between land uses. Homogeneity of multivariate dispersion (PERMDISP) was not tested; this is considered in the Discussion. After quality filtering, library sizes ranged from 13,096 to 47,233 reads per sample. Because richness estimators depend strongly on sequencing depth (Weiss et al. 2017), all samples were normalized to the smallest library size, so no sample was excluded and all 180 samples were retained (Supplementary Table S3). Normalization was performed by scaling with ranked subsampling (SRS; Beule and Karlovsky 2020), with Cmin set to the smallest library size (13,096 reads), which, unlike random rarefying, is reproducible and better preserves the relative frequencies of ASVs, including rare ones. As an alternative normalization, alpha diversity was also calculated on data rarefied to 13,096 reads by random subsampling without replacement (seed 913); both approaches gave the same ranking of land uses (Supplementary Table S3; see Results). The R and shell scripts used for all analyses are available at https://github.com/rafaelaguimaraes2/maize-soybean-rotation-microbiome. High-performance computing resources were provided by the High Performance Computing Center (HPCC) at Michigan State University, USA.

### Statistical analyses

**Fig. 1:**
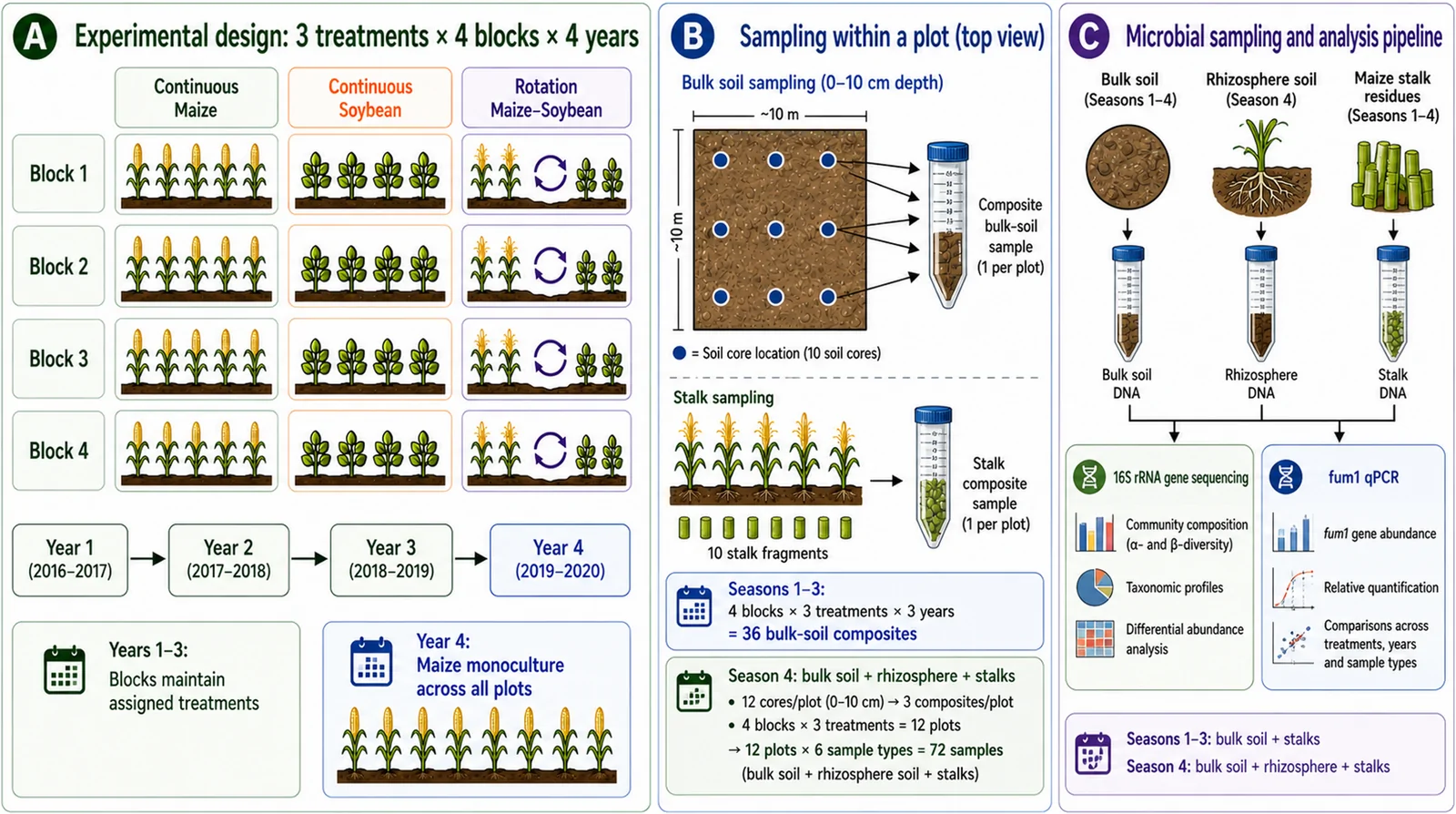
Schematic representation of the experimental layout and soil sampling scheme. One representative block is shown (repeated across four randomized complete blocks; 12 plots total). Soil sampling: 0–10 cm depth. In the first three seasons (2016–2019), bulk soil was sampled at harvest only (10 points combined into 1 composite sample per plot; 36 composite samples total). In the fourth season (2019–2020), both bulk soil and rhizosphere were sampled at blooming (maize tassel stage, VT) and at harvest (12 points combined into 3 composite samples per plot; 72 bulk + 72 rhizosphere composite samples). Total: 180 composite soil DNA samples processed for 16 S rRNA sequencing

**Fig. 2:**
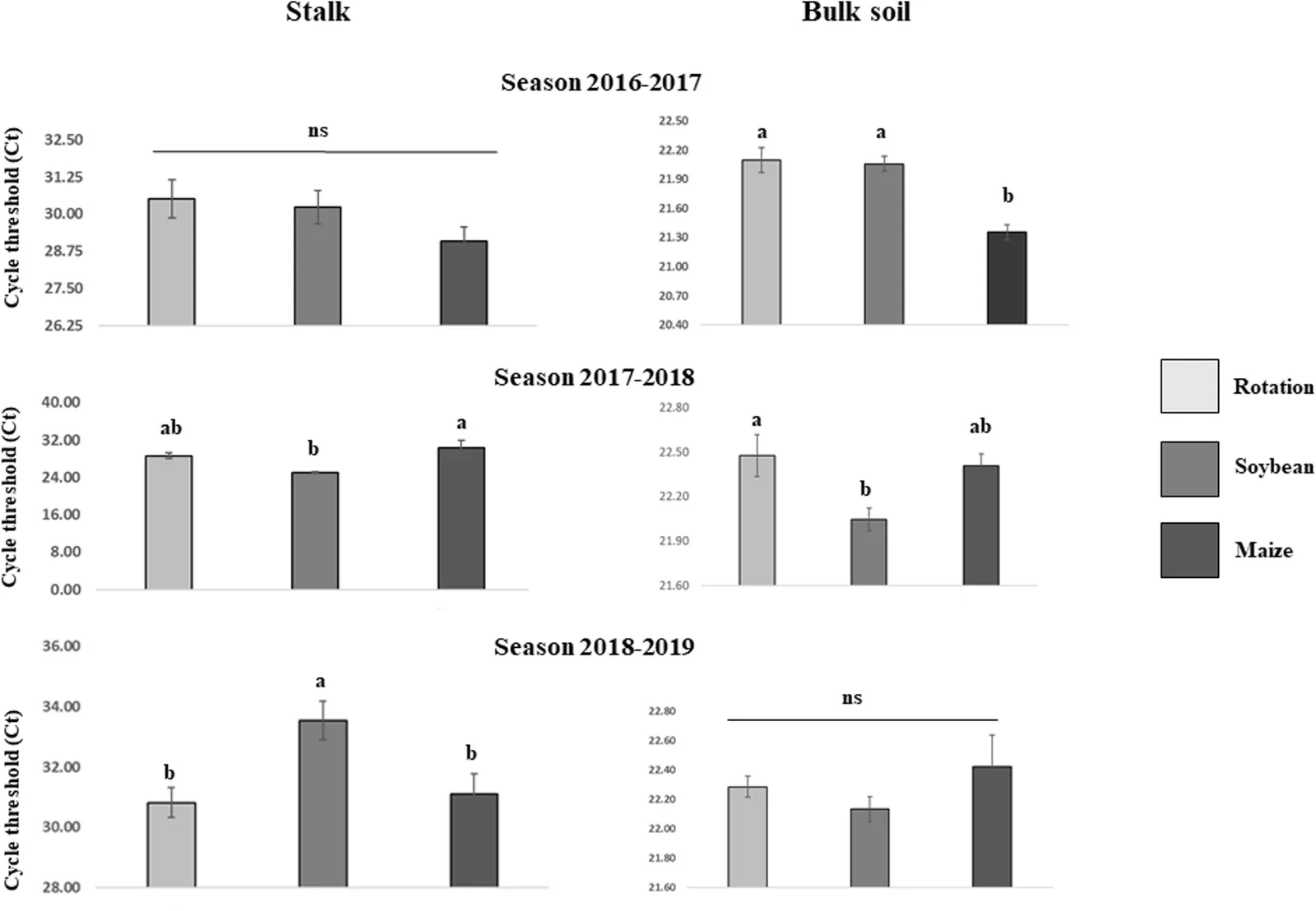
Quantification of *Fusarium* spp. fum1 gene (Ct values) in stalk and bulk soil samples according to land use during seasons 2016–2017, 2017–2018, and 2018–2019 under soybean–maize, soybean, and maize systems. Means were compared by Tukey’s test (*p* < 0.05). Error bars represent the standard error of the mean (SE); *n* = 4 blocks per treatment per season

**Fig. 3:**
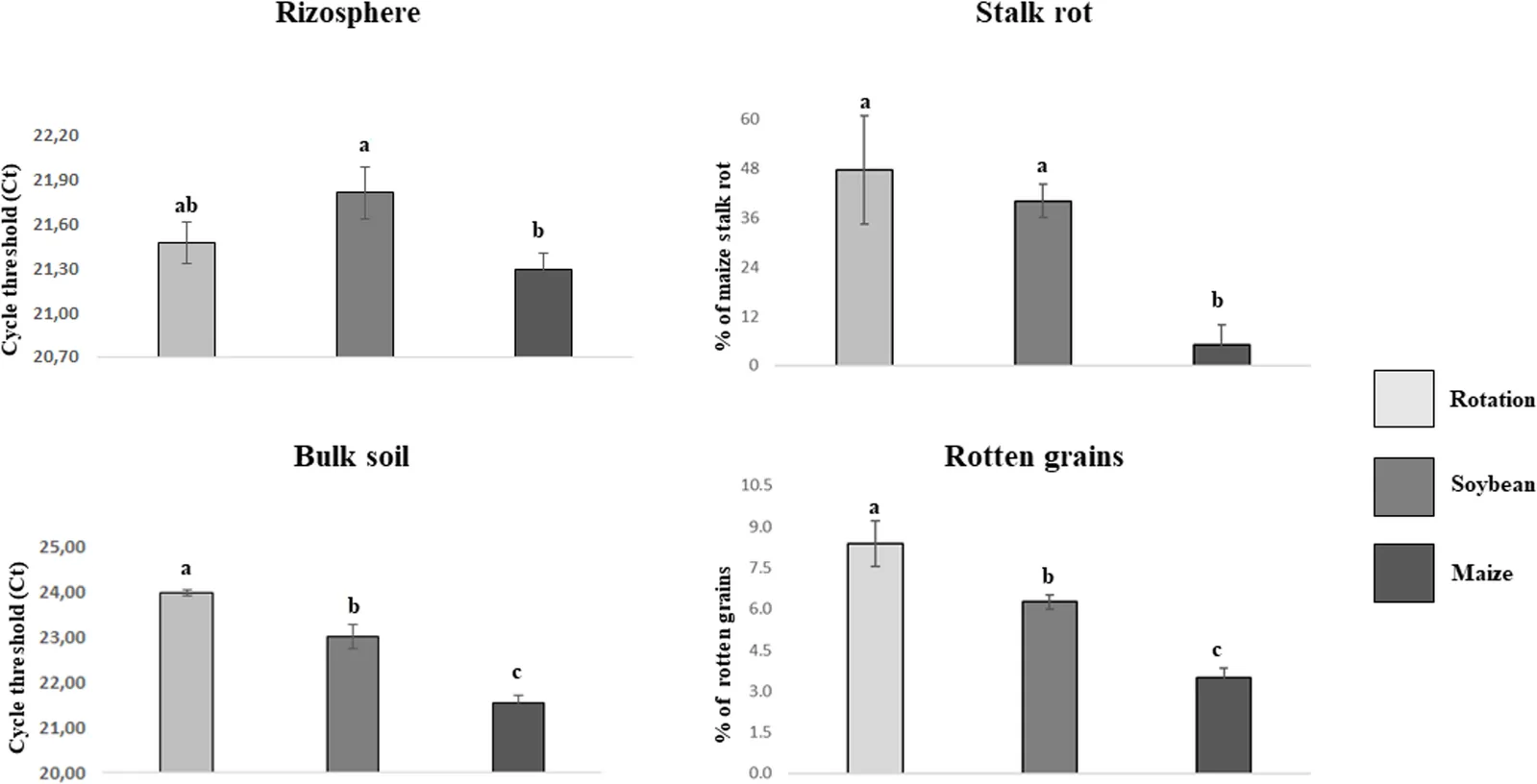
Effect of three-year land uses on *Fusarium* spp. fum1 gene quantification (Ct) in rhizosphere and bulk soil, percentage of stalk rot, and percentage of rotten grains under soybean–maize, soybean, and maize systems. The fourth season was cultivated with maize across all plots. Means were compared by Tukey’s test (*p* < 0.05). Error bars represent the standard error of the mean (SE); *n* = 4 blocks per treatment per season

For all field analyses (except microbiome analyses), normality was assessed with the Shapiro-Wilk test and homogeneity of variances with the F-test. Multiple-comparison tests were performed using Tukey’s HSD at *p* < 0.05, and the Wilcoxon rank-sum test was applied to microbiome diversity comparisons at a significance threshold of *p* < 0.10. For microbiome diversity comparisons (Figs. 1, 2 and 3), the Kruskal-Wallis test was used as the global (omnibus) test across the three land-use treatments, followed by pairwise Wilcoxon rank-sum tests with Benjamini-Hochberg adjustment for multiple comparisons and results are displayed as compact letter displays. Non-parametric tests were used uniformly for these comparisons because normality and homogeneity of variance could not be reliably confirmed across all treatment groups and seasons, and non-parametric tests do not require these distributional assumptions, avoiding the need for different statistical procedures across cases. For field-level variables (Figs. 2 and 3: fum1 gene quantification, stalk rot incidence, and grain quality), normality and homogeneity of variance were assessed with the Shapiro-Wilk and F-tests, respectively (as stated above), justifying the use of one-way ANOVA followed by Tukey’s HSD, which inherently controls the family-wise error rate for multiple pairwise comparisons.

**Fig. 4:**
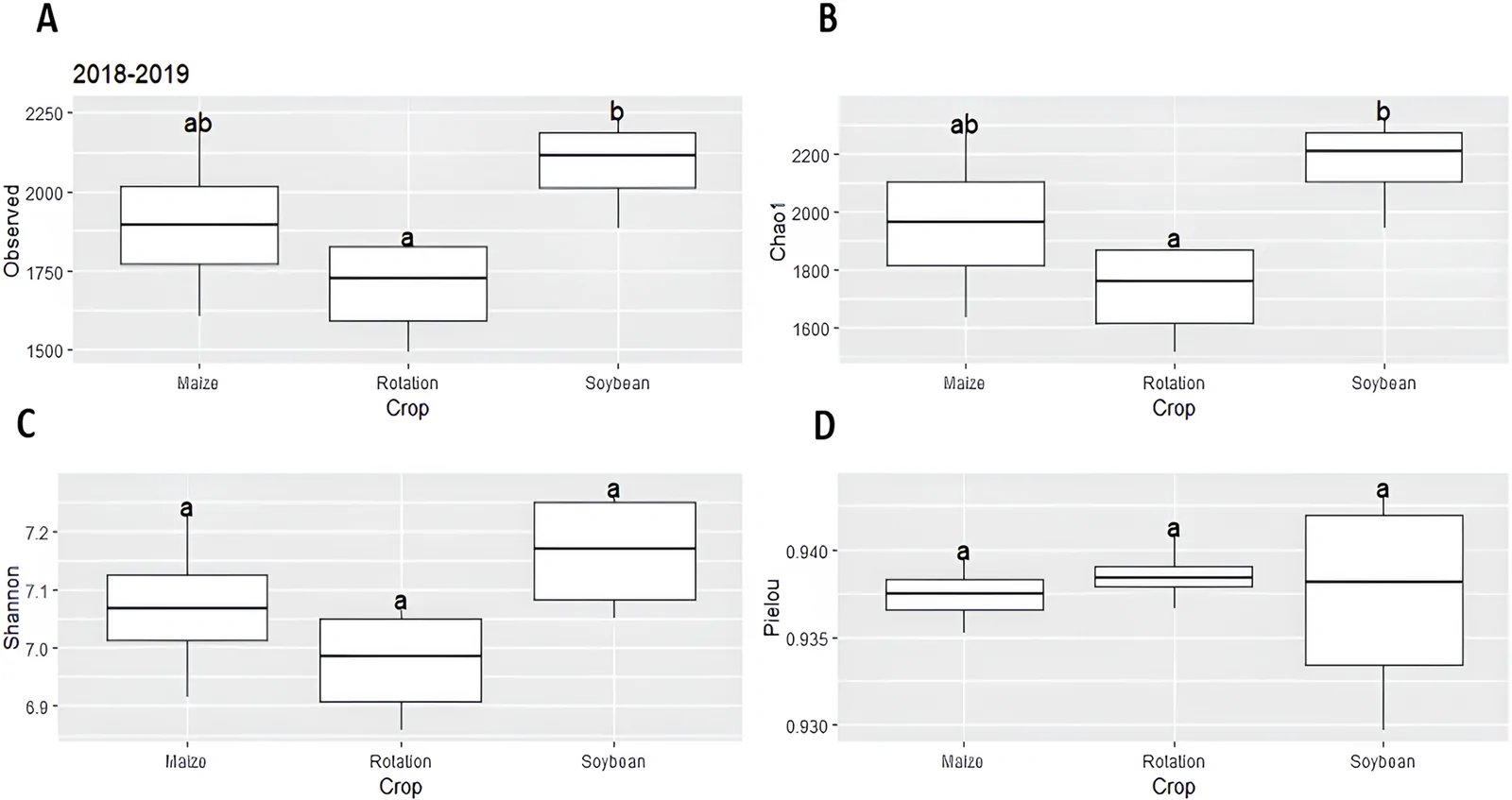
Alpha diversity metrics according to land uses for season 2018–2019. (**A**) Richness (Chao1); (**B**) observed number of taxa; (**C**) Pielou’s evenness; (**D**) Shannon index. Differences were tested using the Wilcoxon rank-sum test (*p* < 0.10). Rotation = annual soybean. Boxplots show median and interquartile range (IQR); *n* = 4 composite samples per treatment. Exact sample sizes, means, standard deviations, and Kruskal–Wallis test statistics (χ², df = 2) and p-values, along with pairwise Wilcoxon p-values, are provided in Supplementary Table S3

Statistical analyses were performed using SigmaPlot^®^ v12 (Systat Software, San Jose, CA, USA). For field-level variables (Figs. 2 and 3), error bars represent the standard error of the mean (SE), with *n* = 4 blocks per treatment per season. For microbiome diversity indices (Figs. 4, 7, and 8), boxplots display the median and interquartile range (IQR), with *n* = 4 composite samples per treatment for the first three seasons and *n* = 12 for the fourth season, reflecting the increased replication described in the “Results” section. For the fourth season, the three composite samples collected per plot were analyzed as separate observations (*n* = 12) rather than averaged, in order to characterize within-plot heterogeneity; however, we note that the field plot (block × treatment), not the composite sample, remains the true experimental unit, as the three composites originate from the same plot and are not independent biological replicates. No mixed-effects or nested model was used for these comparisons; the Kruskal-Wallis and pairwise Wilcoxon tests were applied directly to the composite-sample values, pooled across blocks within each treatment, without a random effect for plot or block. Consequently, the effective sample size for treatment-level inference is bounded by the number of independent plots (*n* = 4 per treatment) rather than by the number of composite samples tested (*n* = 12 in the fourth season), and results should be interpreted with this distinction in mind. Exact sample sizes, means, standard deviations, and Kruskal-Wallis test statistics (χ², df = 2) and p-values, along with pairwise Wilcoxon p-values, for all diversity comparisons are provided in Table S3.

## Results

### Analytical approach

Significance was assessed at *p* ≤ 0.05 for field-level variables analyzed by Tukey’s HSD (Figs. 2 and 3), and at *p* ≤ 0.10 for microbiome diversity and community-structure comparisons analyzed by Kruskal-Wallis/Wilcoxon tests (Figs. 4, 7, 8 and 9). The *p* ≤ 0.10 is less stringent than the conventional threshold, microbiome differences with 0.05 < *p* ≤ 0.10 are described as marginal and treated as exploratory; exact p-values are given in Table S3. The Wilcoxon-based non-parametric approach was chosen for diversity and community-structure comparisons because normality and homogeneity of variance could not be reliably confirmed across all treatment groups and seasons (see the “Statistical analyses” section), whereas Tukey’s HSD was used for field-level variables for which these assumptions were verified. Results from the first three seasons and the fourth season are presented separately throughout, rather than pooled, for three reasons: (i) replication differed substantially between periods (*n* = 4 composite samples per treatment in seasons 1–3 versus *n* = 12 in season 4, see the “Site description and experimental design” section), so pooling would conflate statistical power with any biological effect; (ii) the fourth season introduced a rhizosphere compartment and a blooming (VT) sampling time point that were not present in the first three seasons, making the sample sets structurally different; and (iii) the two periods correspond to conceptually distinct phases of the experiment, an active three-year rotation phase (seasons 1–3) versus a subsequent single-season maize monoculture used to test the residual, legacy effect of prior land use (season 4), such that combining them into a single comparison would obscure this distinction rather than clarify it.

### Inoculum potential of fumonisin-producing *Fusarium* spp. (fum1) in soil and maize stalks

During the study, we evaluated the effect of different land uses on the concentration of fumonisin gene (fum1) in bulk and rhizosphere soil samples, as well as the incidence of maize stalk rot and grain quality. Fumonisin gene (fum1) abundance was quantified by qPCR and is reported as Ct (cycle threshold) values throughout (Figs. 2 and 3); note that lower Ct values indicate higher fum1/target DNA concentration, and vice-versa. In the first season, plots cultivated with continuous maize had the highest concentration of fum1, whereas rotation (cultivated with annual soybean) and continuous soybean showed the lowest concentrations, with no significant difference between them (Fig. 2, Season 2016–2017). However, in the second season (Fig. 2, Season 2017–2018), fum1 abundance was high (low Ct) under both continuous soybean and continuous maize. There was no significant difference in pathogen survival according to land use in the third season (Fig. 2, Season 2018–2019). In the fourth season (Fig. 3), when maize was planted across the entire area to evaluate residual effects of three-year land uses, a soybean–maize rotation history was associated with lower fum1 abundance (higher Ct) in bulk soil than continuous soybean or continuous maize. However, in rhizosphere soil, fum1 abundance was lower after continuous soybean than after continuous maize, with rotation intermediate (Fig. 3). Quantification of fum1 in maize stalks left on the soil surface showed no difference between land uses in the first season and mostly undetermined Ct values in the second season. In the third season, fum1 abundance in stalks was lower under continuous maize than under rotation (annual soybean) and continuous soybean.

### Maize stalk rot and grain quality

Maize stalk rot (Fig. 3) was similar between rotation and continuous soybean, and lower under continuous maize. Grain health (Fig. 3), assessed by the blotter test, showed a lower percentage of infected/rotten grains under a continuous-maize history (about 3.5%) than under continuous soybean (6.45%) and rotation (8.38%), i.e., about half; all three treatments differed significantly (Fig. 3).

### Bacterial diversity during the three-year rotation phase (seasons 1–3)

**Fig. 5:**
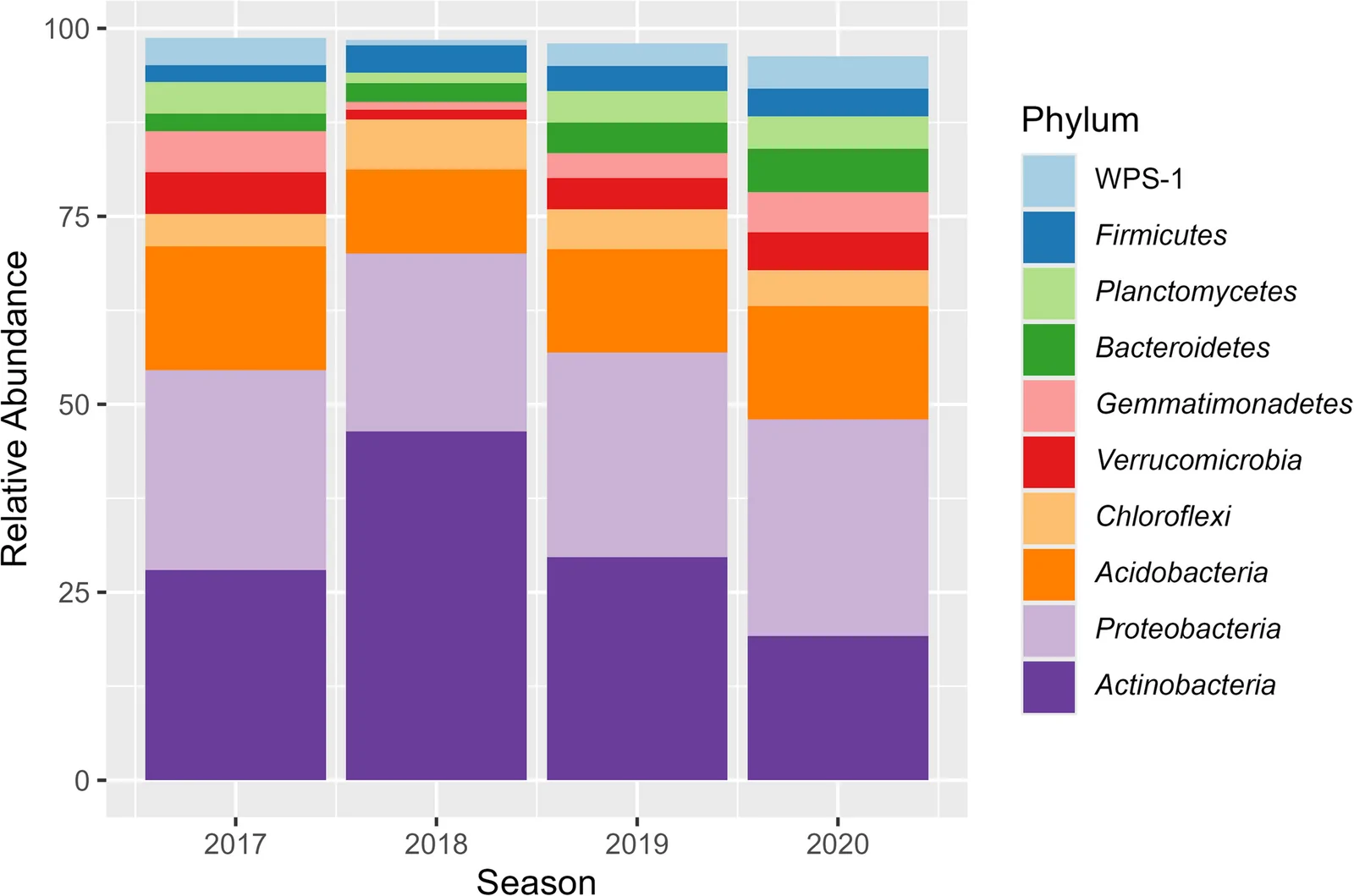
Relative abundance (%) of the top ten phyla according to growing seasons. From left to right: first (2016–2017), second (2017–2018), third (2018–2019), and fourth (2019–2020) seasons

Alpha-diversity metrics, including richness (Chao1), evenness (Pielou’s index), and the Shannon diversity index, did not differ among continuous soybean, continuous maize, and the maize-soybean rotation in bulk soil samples during the first two seasons (Supplementary Fig. S1). In the third season, richness tended to be higher under continuous soybean than under continuous maize and rotation, but the difference was marginal (Kruskal-Wallis, *p* = 0.092) and no pairwise contrast was significant after Benjamini-Hochberg adjustment (Fig. 4; Supplementary Table S3). The same ranking of land uses was obtained with rarefied data. Pielou’s and Shannon indices showed no difference among soil uses (*p* > 0.10). Relative abundance at phylum level showed Actinobacteria, Proteobacteria, and Acidobacteria as the most abundant phyla, representing 71.1%, 81.2%, and 70.7% of all phyla for all seasons at harvest time (Fig. 5). Actinobacteria substantially increased in abundance in the second season, while the minor phyla Verrucomicrobia, Gemmatimonadetes, Bacteroidetes, and WPS-1 decreased (Fig. 5).

### Precipitation as a driver of community structure

**Fig. 6:**
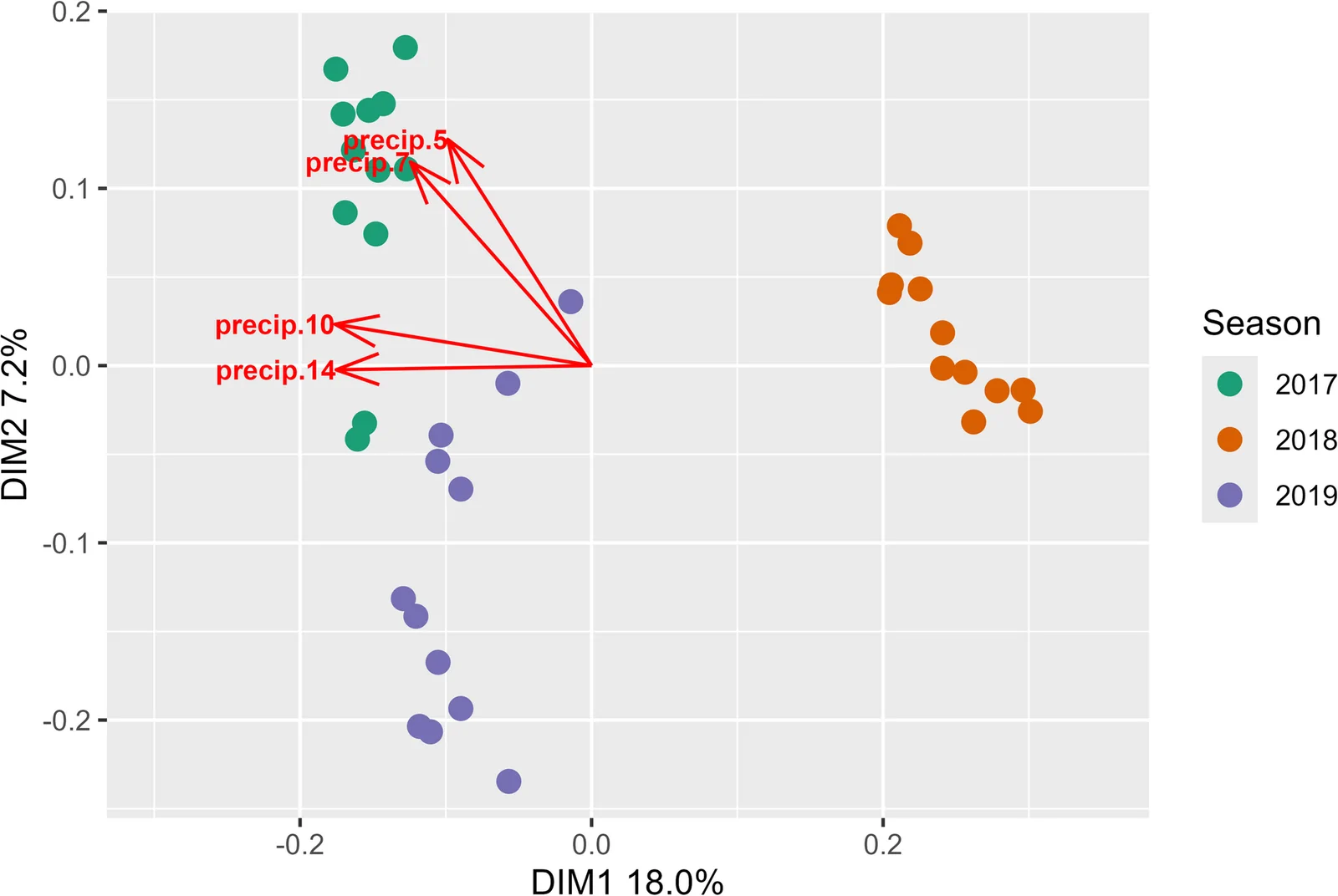
Association between antecedent precipitation (mm) and bulk-soil bacterial community structure (Bray-Curtis PCoA) at 5, 7, 10, and 14 days prior to the sampling time point for three-year land uses. First (2016–2017), second (2017–2018), and third (2018–2019) seasons. Crop rotation land use consisted of annual soybean, annual maize, and annual soybean for the respective three-year experiments

Community structure showed no difference according to three-year soil uses (Supplementary Fig. S2), but differed among seasons, and this seasonal separation was associated with antecedent precipitation (Fig. 6): all four precipitation windows (5, 7, 10 and 14 days) were significantly fitted to the ordination (*p* ≤ 0.05). The precipitation varied only among seasons, its effect cannot be separated from other season-related factors. Results showed higher similarity between communities from the first and third seasons, which were very distinct from the second (Fig. 6). Mean precipitation for 5, 7, 10, and 14 days prior to sampling ranged from 0.2 to 3 mm in the driest year (second season), while it ranged from 3.2 to 123 mm for the first and third seasons.

### Effect of three-year land use on rhizosphere and bulk soil diversity (fourth season)

**Fig. 7:**
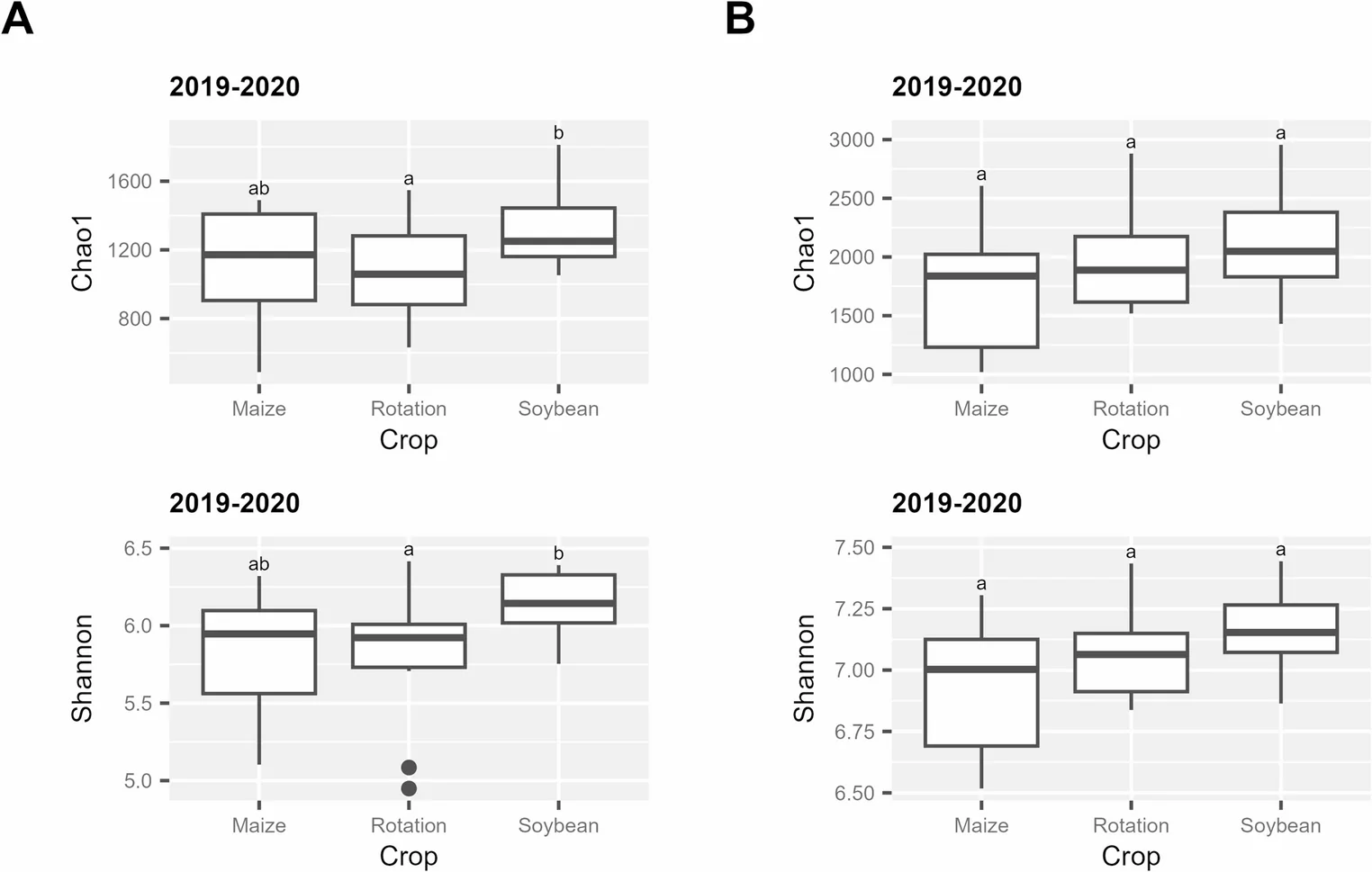
Effect of three-year land uses on alpha diversity metrics at blooming (maize tasseling) time point in (**A**) maize rhizosphere and (**B**) maize bulk soil. Differences were tested using the Wilcoxon rank-sum test (p < 0.10). The fourth season was cultivated with maize across all plots. Boxplots show median and interquartile range (IQR); n = 12 composite samples per treatment. Exact sample sizes, means, standard deviations, and Kruskal-Wallis test statistics (χ², df = 2) and p-values, along with pairwise Wilcoxon p-values, are provided in Supplementary Table S3

**Fig. 8:**
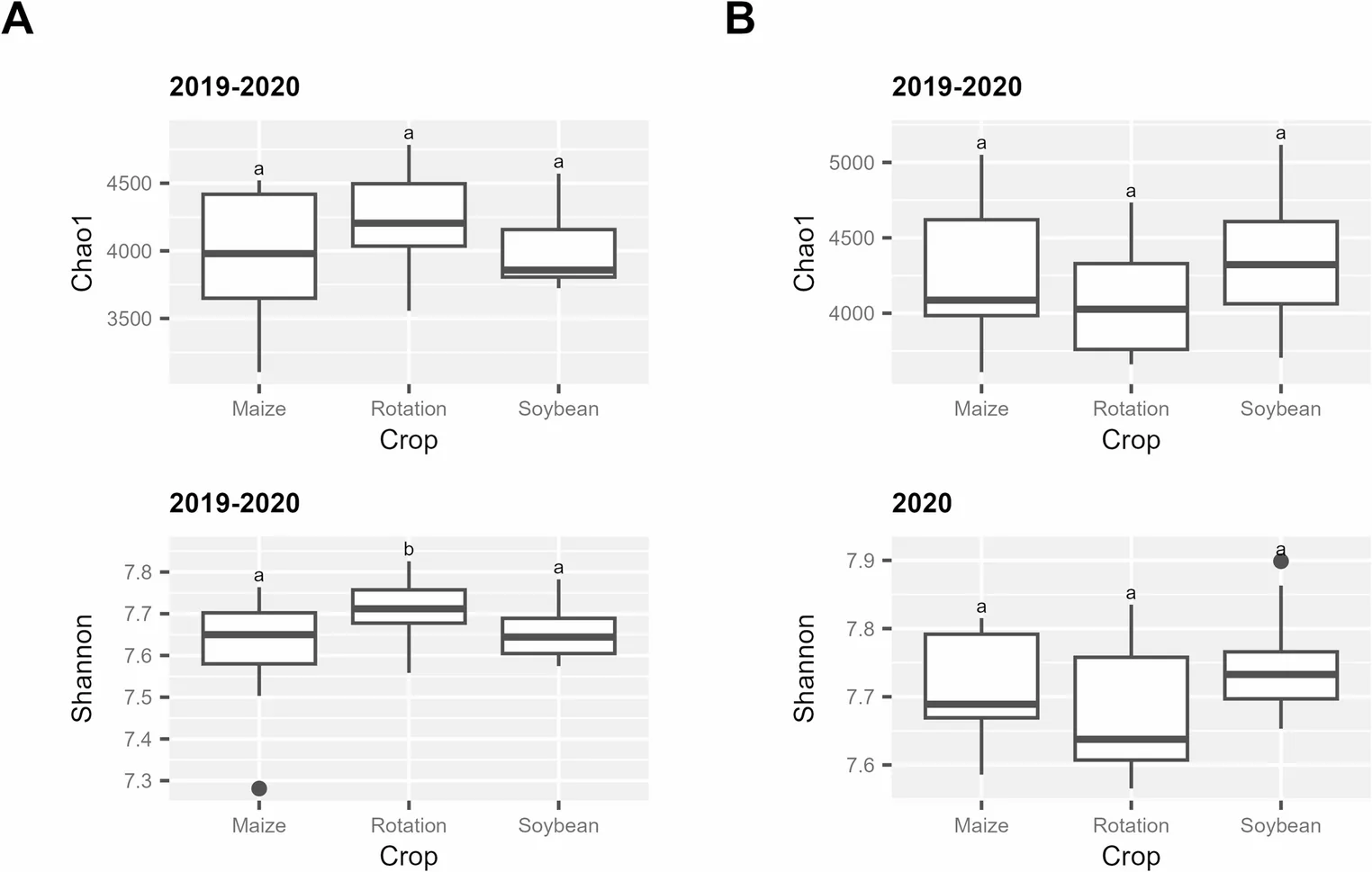
Effect of three-year land uses on alpha diversity metrics at blooming (maize tasseling) time point in (**A**) maize rhizosphere and (**B**) maize bulk soil. Differences were tested using the Wilcoxon rank-sum test (*p* < 0.10). The fourth season was cultivated with maize across all plots. Boxplots show median and interquartile range (IQR); *n* = 12 composite samples per treatment. Exact sample sizes, means, standard deviations, and Kruskal-Wallis test statistics (χ², df = 2) and p-values, along with pairwise Wilcoxon p-values, are provided in Supplementary Table S3

**Fig. 9:**
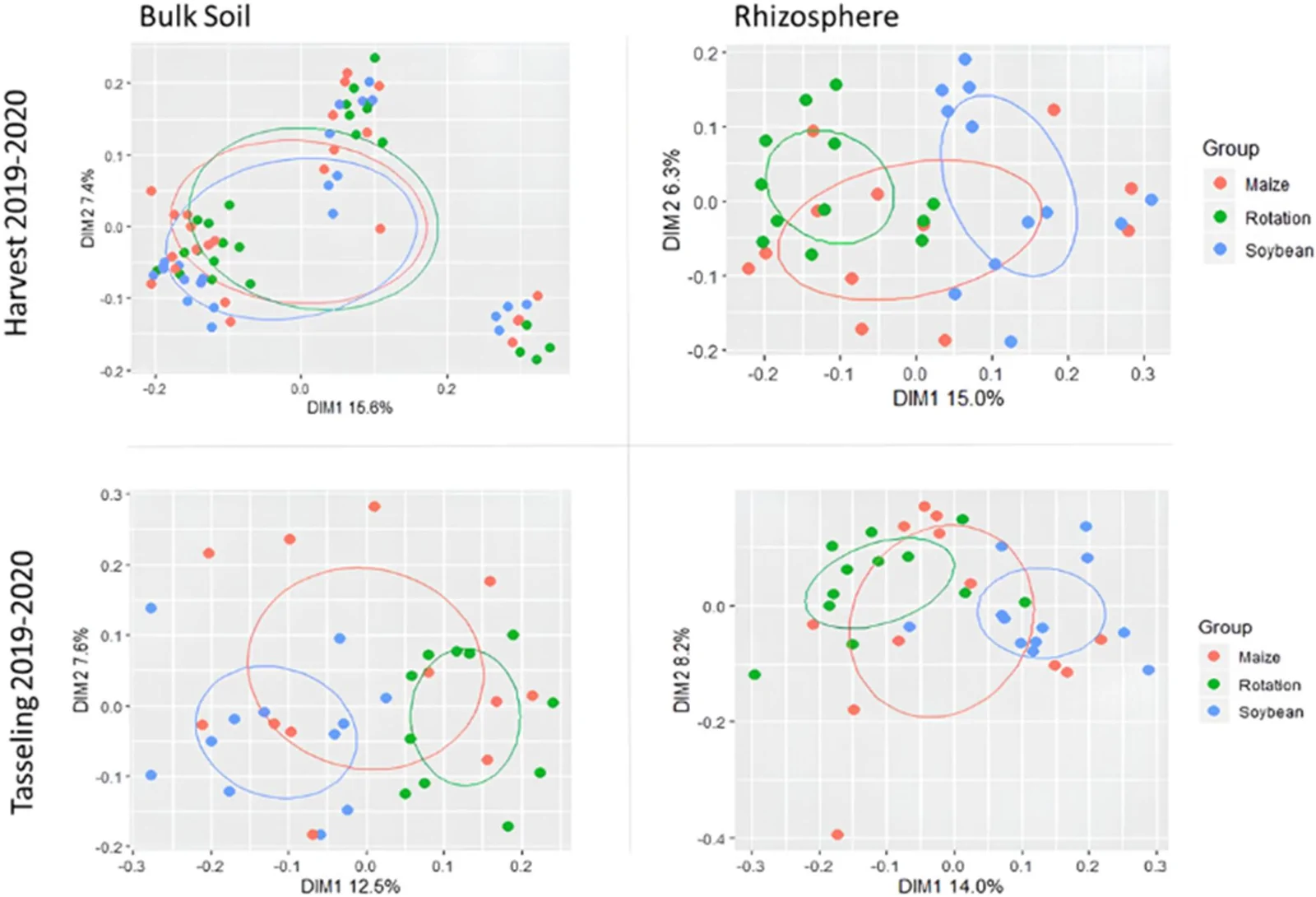
Principal coordinate analysis (PCoA) using Bray-Curtis dissimilarity by land use, bulk soil, and rhizosphere during season 2019-2020 at harvest and maize blooming (maize tasseling). Differences were tested at p ≤ 0.10. Blooming-stage and harvest-stage samples were sequenced in separate runs

To reduce within-plot sampling variability, which may have masked land-use effects in the first three years, sampling intensity was increased in the fourth season to three composite samples per plot (12 composite samples per treatment, from four independent plots). As rhizosphere communities respond faster than bulk soil, we hypothesized that three-year land uses could affect maize rhizosphere communities in the fourth year, visible at both blooming and harvest. A three-year continuous-soybean history was associated with higher richness and diversity of bacterial communities in the maize rhizosphere during blooming of the fourth year (Fig. 7A), although bulk soil was not affected (Fig. 7B). At harvest, rhizosphere Shannon diversity was slightly higher after rotation than after continuous maize or continuous soybean (Kruskal-Wallis *p* = 0.079; pairwise *p* = 0.089), whereas bulk-soil diversity did not differ among land uses (Fig. 8; Supplementary Table S3). Beta diversity (Bray-Curtis dissimilarity) showed that three-year soil uses did not affect bulk soil community structure at harvest (*p* > 0.10), but in the rhizosphere, continuous soybean and rotation showed the most dissimilar structures (*p* = 0.003) compared to continuous maize–continuous soybean or continuous maize-rotation (*p* = 0.032) (Fig. 9). At blooming, community structure differed among land-use histories in both bulk soil and rhizosphere (PERMANOVA, *p* < 0.05) (Fig. 9).

### Relative abundance of dominant phyla in the fourth season

Proteobacteria, Actinobacteria, Bacteroidetes, and Firmicutes were the most abundant phyla in rhizosphere samples at both time points. Proteobacteria, Bacteroidetes, and Firmicutes increased their relative abundances by 6.3%, 10.3%, and 5.2% at blooming compared to harvest, whereas Acidobacteria increased by 7.2% at harvest (Table 1). Bulk soil samples were similar in abundance at both sampling times, with the exception of Actinobacteria, which represented 27% of phyla at blooming compared to 19.2% at harvest (Table 1).

**Table 1:** Mean relative abundance (%) of the ten most abundant phyla in the rhizosphere and bulk soil at blooming (maize tasselling) and harvest time points for the 2019–2020 season, when maize was cultivated across the whole experimental area

Phylum	Rhizosphere Blooming	Rhizosphere Harvest	Bulk soil Blooming	Bulk soil Harvest
Proteobacteria	41.5	35.2	28.6	28.8
Actinobacteria	17.7	18.7	27.0	19.2
Bacteroidetes	19.1	8.84	3.79	5.77
Acidobacteria	4.62	11.8	12.6	15.1
Firmicutes	7.98	2.79	4.38	3.70
Chloroflexi	1.91	4.76	5.82	4.74
Verrucomicrobia	2.19	3.80	4.65	5.07
WPS-1	1.75	3.54	3.11	4.33
Gemmatimonadetes	1.37	3.58	5.32	5.35
Planctomycetes	1.17	3.58	2.90	4.27

Values are mean relative abundances (%) of bacterial 16 S rRNA gene sequences

## Discussion

Soil biodiversity plays a crucial role in maintaining soil quality, promoting plant health, and improving resilience against biotic and abiotic stresses. Microbial communities are responsible for several processes in the edaphic ecosystem (Nielsen et al. 2015). However, in agroecosystems, intensive land use, simplified crops, conventional planting systems, and the indiscriminate use of chemical fertilizers and pesticides have been found to decrease soil biodiversity, functionality, and plant yield, making them more susceptible to plant pathogens and pests (Tsiafouli et al. 2015; Allan et al. 2015; Soares de Carvalho et al. 2016). Previous studies have suggested that crop rotation, cover crops, and no-tillage can be used as alternatives to these intensive practices and as allies to build soil suppression to pathogens (Tiemann et al. 2015; Peralta et al. 2018; Li et al. 2025). A three-year crop rotation between maize and soybean was hypothesized to increase microbial diversity in soil and decrease the inoculum potential of fumonisin-producing *Fusarium* spp. (fum1) in soil and maize stalks. However, rotation did not significantly alter bacterial alpha diversity in the first two seasons, although the power to detect moderate effects was low (see below).

In the third season (2018–2019), richness tended to be higher under continuous soybean than under continuous maize and rotation (annual soybean), but this trend was not significant after adjustment for multiple comparisons; evenness and Shannon diversity did not differ. A recent study found that rotational maize-soybean or continuous cropping did not affect richness and diversity in bulk soils; however, communities from continuous maize and continuous soybean showed greater dissimilarity, while this did not occur in rotational systems (Chamberlain et al. 2020). The mayze-soybean rotation with a rye cover crop found that rotation and cover cropping had only minor, inconsistent effects on soil bacterial and fungal community composition relative to soybean yield outcomes, further supporting the view that maize-soybean rotation effects on soil microbial communities are often subtle and context-dependent rather than uniformly large (Frey et al. 2025).

The absence of a detectable rotation effect on bacterial alpha diversity should be interpreted in light of the rotation itself. A meta-analysis showed that crop rotation increases bacterial Shannon diversity and richness only modestly and does not affect bacterial beta diversity; these effects were larger with more rotational cycles and longer durations, and the response of bacterial richness was greater in rotations lasting 10–30 years than in those lasting 5 years or less (Li et al. 2025). Our rotation included only two crops, lasted three seasons and was established on an area with ten years of maize monoculture, placing it at the lower end of these gradients. Even much longer contrasts may leave alpha diversity unchanged: after 55 years of rye and maize monocropping, bacterial alpha diversity did not differ from that under crop rotation, although community composition did (Gryta et al. 2026). This pattern, in which composition responds more than diversity, agrees with our observation that land-use history was detected mainly in community structure and in the rhizosphere. The same meta-analysis also showed that transitions from a legume to a non-legume crop increase bacterial Shannon diversity more than the reverse transition (Li et al. 2025), which is consistent with the higher rhizosphere diversity of maize grown after continuous soybean at tasselling in the fourth season. In addition, all treatments were under no-till with residues retained, which may buffer short-term differences in bulk soil, and four plots per treatment provide limited power to detect small effects. Functional redundancy among soil bacteria may also allow taxonomic turnover without changes in diversity indices.

Land-use history affected the rhizosphere more than bulk soil, suggesting that crop history acts mainly through the root-associated community. The root holobiont is centred on a host-specific core microbiome, whose assembly depends largely on root-secreted metabolites and the chemotactic response of soil bacteria to them (Gupta et al. 2025). Future work should therefore identify the core rhizosphere microbiota of maize and soybean and determine how it differs from, and is recruited from, the bulk-soil community, to better understand holobiont dynamics under rotation and monoculture. Identifying core taxa alone, however, cannot reveal the mechanisms involved. Root exudates differ between maize and soybean (Pascale et al. 2020), and long-term monocropping has been associated with the accumulation of root-derived phenolic compounds in soil (Gryta et al. 2026). Core-microbiome analyses should therefore be combined with root-exudate and rhizodeposit metabolomics, so that shifts in microbial communities can be linked to the compounds that drive them.

We found no significant effect of land use on bulk-soil community structure in the first three seasons, whereas community structure differed among seasons in association with antecedent precipitation. Because precipitation varied only among three seasons, this association is correlative and cannot be separated from other season-related factors (e.g., temperature, sampling date, crop phase); precipitation is therefore best regarded as a likely contributor rather than an established driver. The taxonomic profile and abundances of microbes were similar across seasons, except for a 21% increase in Actinobacteria during the second and driest season compared to other seasons. On the other hand, the abundances of Verrucomicrobia, Gemmatimonadetes, Planctomycetes, and WPS-1 were lower in the driest season (4.5%) compared to a mean of 17.5% for seasons with higher precipitation. Our findings are consistent with other studies that observed stimulation of Actinobacteria under dry conditions and a reduction under rewetting in forest soils (Zhou et al. 2016). Actinobacteria are likely r-strategists, characterized by fast-growing and highly variable populations (Ho et al. 2017). Tan et al. (2020) found that mean annual precipitation and soil pH were the major drivers shaping microbial communities in maize soils. In the same study, the most abundant phyla were Proteobacteria, Actinobacteria, Acidobacteria, Chloroflexi, and Gemmatimonadetes; with increasing mean annual precipitation, the relative abundance of Proteobacteria increased while Gemmatimonadetes and Nitrospirae decreased. Gemmatimonadetes are frequently found in soil microbiomes and seem to be more adapted to low moisture conditions (De Bruyn et al. 2011). Planctomycetes have been found in a range of environmental conditions, including marine and freshwater (Buckley et al. 2006), as well as Verrucomicrobia (Freitas et al. 2012).

We analyzed precipitation data from 14, 10, 7, and 5 days prior to the sampling time point for each season. Communities from the first and third seasons were clearly separated from those of the second and driest season (2017–2018). During the 14 days prior to sampling, average precipitation for the first and third seasons ranged from 120 to 123 mm, while the second season had little to no precipitation (0–3 mm). Environmental factors play a crucial role in microbial community assembly. Soil pH (Soares de Carvalho et al. 2016; Tan et al. 2020), organic carbon (Davey et al. 2019), and precipitation (Zhou et al. 2016; Tan et al. 2020) have been identified as primary drivers.

In the fourth season (2019–2020), we evaluated the impact of three-year land uses on maize bulk soil and rhizosphere diversity during blooming and harvest, as well as the effect of rotation and continuous cropping on fum1 inoculum concentration and maize stalk and ear rot incidence. Our findings revealed that three-year land uses had no effect on bulk soil diversity (richness and Shannon index) during maize blooming. However, the rhizosphere of maize grown in soils with a continuous-soybean history showed higher ASV richness and Shannon diversity than that of maize grown after rotation (*p* < 0.10), but did not differ from that after continuous maize. At harvest, by contrast, rhizosphere Shannon diversity was slightly higher after rotation, and bulk-soil diversity did not differ among land uses. Thus, land-use history affected alpha diversity mainly in the rhizosphere, and the direction of the effect differed between tasselling and harvest, rather than being consistent across compartments and time points. Plants select microorganisms in their immediate environment based on factors such as their genetic makeup and physiological state (Peiffer and Ley 2013; Mendes et al. 2014).

Soybeans are legumes that have complex relationships with soil microorganisms, including nitrogen-fixing bacteria in *Bradyrhizobium* spp., as well as *Bacillus* and *Paenibacillus* (Martínez-Hidalgo and Hirsch 2017). Plants affect microbiome composition by releasing metabolites and sugars, which differ between maize and soybean (Kudjordjie et al. 2019; Pascale et al. 2020). Zhou et al. (2018) found that legume systems have greater diversity than grass systems and support more fungi. Although only maize was planted in the fourth year, soybean residues and root systems may still have influenced the soil microbiome.

In the fourth season, land-use history was associated with differences in community structure in both compartments at tasselling and in the rhizosphere at harvest, but not in bulk soil at harvest; the largest difference was between continuous-soybean and rotation histories in the rhizosphere at harvest. Although crop rotation has been linked to greater diversity in agroecosystems, this has been associated with more diversified rotational systems, such as the five-crop rotation studied by Tiemann et al. (2015). Comparisons between the first three seasons and the fourth season confound statistical power with the biological legacy effect of land use, since replication increased from *n* = 4 (blocks) to *n* = 12 (blocks × composite subsamples) specifically in the fourth season. A post-hoc power analysis (Wilcoxon rank-sum test) showed that even the largest pairwise difference detected in the study (Chao1, continuous soybean vs. rotation, Cohen’s d = 2.30) had only 61% power to reach significance at α = 0.05 with *n* = 4, and would have required d ≈ 3.0 to reach conventional 80% power at this sample size. Under the same effect sizes, replication of *n* = 12 yielded power ≥ 99%, although this assumes 12 independent observations, which the composite samples are not. This demonstrates that the first three seasons were underpowered to detect effects of this magnitude. However, because post-hoc power calculated from observed effect sizes is mathematically related to the obtained p-values, this analysis cannot itself establish that a true biological effect was present but undetected in those seasons; it shows only that those seasons had limited ability to detect one. We therefore interpret the higher diversity detected under continuous soybean history in the fourth season as consistent with, rather than newly caused by, the higher replication achieved that year, without concluding that an equivalent effect was necessarily present earlier. This increase from *n* = 4 to *n* = 12 reflects greater subsampling intensity within each plot in the fourth season, not additional independent biological replication, and should be interpreted accordingly.

Regarding a potential precipitation confound in the fourth season (Suplementaru Table S1), available data indicate that the blooming-stage sampling point (28 January 2020) occurred under a moderate-to-wet regime (62.7 mm over the preceding 14 days, with 10 of 14 days recorded), more comparable to the wetter first and third seasons (120–123 mm/14 days) than to the anomalously dry second season (3 mm/14 days), which argues against precipitation as the main driver of the blooming-stage differences attributed to land-use legacy (Figs. 7 and 9, blooming). For the harvest-stage sampling point (19 April 2020), however, precipitation records remain largely unavailable (5 of 14 days), and a precipitation confound cannot be ruled out for these specific comparisons (Figs. 8 and 9, harvest); this should be considered when interpreting the harvest-stage results.

A further limitation of this study is that bacterial diversity, assessed via 16 S rRNA sequencing, is an indirect proxy for suppression of *F. verticillioides*, a fungal pathogen. Fungal community profiling (e.g., ITS sequencing) was not performed in this study, limiting our ability to draw direct mechanistic links between the observed bacterial community shifts and fum1 suppression. Future studies incorporating fungal community profiling alongside differential abundance or indicator-taxa analyses (e.g., ANCOM-BC, DESeq2) of the bacterial community could help identify candidate bacterial taxa directly associated with reduced fum1 abundance, strengthening the mechanistic basis for the suppression patterns reported here.

A further limitation concerns the sequencing-batch structure of the fourth season. Blooming-stage samples were processed in the first sequencing run, whereas harvest-stage samples were processed in a separate, second run. Because Fig. 9 displays blooming- and harvest-stage samples together in the same ordination, the separation observed between these two time points may partly reflect a technical batch effect in addition to genuine phenological or biological differences, and this should be considered when interpreting the blooming-versus-harvest separation in Fig. 9. All within-time-point statistical comparisons among land-use treatments (Figs. 6, 7 and 8), by contrast, are unaffected by this batch structure, as each draws exclusively on samples from a single sequencing run.

This study was conducted at a single field site, selected specifically for its confirmed long-term history of *F. verticillioides* incidence, which was necessary to test rotation-driven pathogen suppression under naturally high inoculum pressure. While the four-season temporal design captured substantial inter-annual environmental variation, the findings should be interpreted with caution when extrapolating to other soils, climates, or cropping histories, and future multi-location trials would help establish the generality of the rotation-legacy effects reported here.

Additional limitations concern the pathogen data. fum1 abundance was reported as Ct values, which were not converted to absolute quantities or normalised to soil mass or DNA input, and amplification efficiency varied among runs; comparisons were therefore restricted to treatments within the same season and compartment. The qPCR detects DNA from both viable and non-viable propagules, so fum1 abundance indicates inoculum potential rather than viable inoculum, and the presence of fum1 does not demonstrate fumonisin production, which was not measured. Finally, the fourth-season beta-diversity comparisons, like the alpha-diversity comparisons, used composite samples nested within plots; effects detected only at this level should be confirmed with greater plot-level replication. In addition, homogeneity of multivariate dispersion was not tested, so part of the PERMANOVA differences may reflect differences in within-group variability rather than in community location. Finally, the time between sampling and DNA extraction differed among seasons, which may have contributed to the differences between seasons, including the shift associated with precipitation, but not to differences among land uses within a season.

Throughout the four seasons, we analyzed the behavior of *Fusarium* spp. fum1 in soil and maize stalks, and evaluated disease incidence influenced by three-year land uses. Even though other *Fusarium* spp. fumonisin producers, such as *F. proliferatum*, exist in maize, *F. verticillioides* is responsible for the majority of maize ear and stalk rots in tropical regions (Stumpf et al. 2013). In bulk soil, *Fusarium* spp. fum1 abundance was lower under rotation than under continuous maize in the first, second and fourth seasons. However, in the first season the rotation plots were cropped with soybean and the effect was shared with continuous soybean, and in the second season they were cropped with maize; these differences therefore reflect, at least partly, the crop present in each season rather than the rotation sequence itself. Only the fourth-season difference, when all plots were cropped with maize, can be interpreted as a legacy of land-use history.

In the rhizosphere, fum1 abundance was lower after continuous soybean than after continuous maize, with rotation intermediate. Interestingly, in the second year (2017–2018), fum1 abundance in soil was also high under continuous soybean. Some studies suggest that at higher concentrations, *F. verticillioides* can affect soybean seed germination (Pedrozo and Little 2017). However, it is not clear whether soybean is a secondary host for this pathogen. Continuous maize was associated with higher fum1 abundance in soil in most seasons. On the other hand, when analyzing maize stalks left on the soil surface, fum1 abundance was lower under continuous maize in the third season, and a continuous-maize history was associated with lower stalk-rot incidence and fewer infected grains in the fourth season. These observations are consistent with, but do not demonstrate, the development of residue-associated suppression after repeated maize cropping; alternative explanations, including differences in residue decomposition, in the composition of the fumonisin-producing community detected by fum1, or chance given four plots per treatment, cannot be excluded. Maize stubble is the major source of pathogen multiplication (Munkvold 2003; Blacutt et al. 2018). Crop residues can harbour antagonistic microorganisms (Kohl et al. 2019), but whether maize stalks become suppressive after consecutive maize cropping remains to be tested, for example with residue-based suppressiveness bioassays combined with fungal community profiling.

## Conclusions

Under the limited replication achieved in the first three seasons (*n* = 4; post-hoc power of 12–61% for the observed effect sizes), rotation did not significantly enhance soil bacterial diversity compared to continuous cropping, a result that should be interpreted with caution given this power limitation. Seasonal differences in bulk-soil communities were associated with antecedent precipitation, although this association is correlative. With greater sampling intensity in the fourth season, a continuous-soybean history was associated with higher bacterial diversity in the maize rhizosphere at tasselling; because these comparisons rely on composite samples nested within four plots per treatment, this finding should be confirmed with greater plot-level replication. Rotation did not detectably increase bacterial diversity, but a rotation history was associated with lower abundance of fumonisin-producing *Fusarium* spp. (fum1) in bulk soil in the fourth season. In contrast, continuous maize was associated with lower fum1 abundance in maize stalks (third season only) and with less stalk rot and fewer infected grains in the fourth season, a pattern that may indicate residue-associated suppression but requires direct testing. These results, obtained at a single site, suggest that soil and crop residues respond differently to rotation and that pathogen survival in residues may need to be managed alongside rotation in tropical maize systems; multi-site studies with greater plot-level replication and fungal community profiling are needed to confirm them.

## Supplementary information

Below is the link to the electronic supplementary material.

Supplementary Material 1

## Data Availability

No datasets were generated or analysed during the current study.

## Declarations

### Use generative AI or AI-assisted technology

During the preparation of this work the author(s) used Claude and Skywork in order to improve and edit English. After using this tool/service, the author(s) reviewed and edited the content as needed and takes full responsibility for the content of the published article.
