# Supplementary material for: Effects of a three-year maize-soybean rotation on soil bacterial diversity and fumonisin-producing Fusarium spp. inoculum potential
Source: World J Microbiol Biotechnol. 2026 Oct 20;42(11):616. doi: 10.1007/s11274-026-05358-2 (PMC13683261; doi:10.1007/s11274-026-05358-2)
Supplement: Supplementary file 1 [file 11274_2026_5358_MOESM1_ESM.docx]

**Effects of a three-year maize-soybean rotation on soil bacterial diversity and fumonisin-producing *Fusarium* spp. inoculum potential**

Kize Alves Almeida¹, Rafaela Araújo Guimarães¹, Victor Biazzotto Correia Porto¹, Luiz Flávio Machado Góes¹, Fatima Maria de Souza Moreira², John Quensen³, James Tiedje³, Flávio Henrique Vasconcelos de Medeiros¹

¹ Department of Phytopathology, Laboratory of Biological Control of Plant Diseases, Universidade Federal de Lavras (UFLA), Lavras, 37200-000, MG, Brazil

² Department of Soil Science, Sector of Biology, Microbiology and Biological Processes, Universidade Federal de Lavras (UFLA), Lavras, 37200-000, MG, Brazil

³ Department of Plant, Soil and Microbial Sciences, Center for Microbial Ecology, Michigan State University, East Lansing, MI, 48824, USA

**Corresponding authors:**

Rafaela Araújo Guimarães, Department of Phytopathology, Universidade Federal de Lavras (UFLA), Lavras, 37200-000, MG, Brazil. E-mail: rafaela.guimaraes3 @ufla.br

Flávio Henrique Vasconcelos de Medeiros, Department of Phytopathology, Universidade Federal de Lavras (UFLA), Lavras, 37200-000, MG, Brazil. E-mail: flaviomedeiros@ufla.br

**Supplementary Material**

**Supplementary Figures**

**Figure S1** Alpha diversity metrics showed by Richness (Chao1), observed number of taxa, Pielou’s evenness and Shannon index according to land uses for season A) 2016-2017 and B) 2017-2018. Differences were tested by the Kruskal–Wallis test followed by pairwise Wilcoxon rank-sum tests with Benjamini–Hochberg adjustment (p < 0.10).

**Figure S2** Similarity in soil microbial communities using Bray-Curtis distance according to three-year land uses and seasons pooled.

**Figure S3** Experimental workflow. Cropping sequence of each land use across the four seasons, soil sampling in the land-use phase (seasons 1-3) and in the legacy test (season 4), laboratory analyses including the sequencing run of each sample set, and the data-analysis pipeline.

**Supplementary Table**

**Supplementary Table S1 Precipitation preceding each soil/rhizosphere sampling point across the four growing seasons.**

| **Season** | **Sampling point** | **Date** | **5 d (mm)** | **7 d (mm)** | **10 d (mm)** | **14 d (mm)** | **14-d coverage** |
| --- | --- | --- | --- | --- | --- | --- | --- |
| 1 (2016-2017) | Harvest | 22 Mar 2017 | 82.4 | 82.4 | 82.4 | 122.8 | 14/14 |
| 2 (2017-2018) | Harvest | 12 Apr 2018 | 0.0 | 0.2 | 2.0 | 3.0 | 14/14 |
| 3 (2018-2019) | Harvest | 26 Mar 2019 | 3.2 | 20.2 | 69.3 | 120.3 | 14/14 |
| 4 (2019-2020) | Blooming (tasseling) | 28 Jan 2020 | 33.3 | 47.7 | 62.7 | 62.7 | 10/14 |
| 4 (2019-2020) | Harvest | 19 Apr 2020 | 0.0 | 0.0 | 0.0 | 5.8 | 5/14 |

*Values are cumulative precipitation (mm) for the 5, 7, 10, and 14 days preceding each sampling date, calculated from local station daily records. Data-day coverage (days with a valid record / total days in window) is shown in parentheses for the 14-day window.**Seasons 1–3 values match those reported in the main text (Section 2, Results). For season 4, precipitation records were partially recovered: coverage was reasonable for the blooming sampling point (10/14 days) but largely unavailable for the harvest sampling point (5/14 days), consistent with a gap in local data collection during March–April 2020.

**Supplementary Table S2 Soil physicochemical properties by land-use treatment**

| **Parameter** | **Rotation** | **Continuous soybean** | **Continuous maize** | **Overall (n=12)** |
| --- | --- | --- | --- | --- |
| pH (H₂O) | 5.78 ± 0.29 | 5.60 ± 0.42 | 5.78 ± 0.29 | 5.72 ± 0.32 |
| P (mg/dm³) | 14.18 ± 2.55 | 15.05 ± 1.85 | 15.38 ± 1.51 | 14.87 ± 1.90 |
| K (mg/dm³) | 331.88 ± 10.77 | 362.60 ± 22.05 | 368.75 ± 42.55 | 354.41 ± 30.69 |
| Ca (cmolc/dm³) | 4.65 ± 0.21 | 5.40 ± 0.48 | 4.80 ± 0.36 | 4.95 ± 0.47 |
| Mg (cmolc/dm³) | 1.10 ± 0.08 | 1.55 ± 0.24 | 1.30 ± 0.36 | 1.32 ± 0.30 |
| Al (cmolc/dm³) | 0.05 ± 0.06 | 0.03 ± 0.05 | 0.08 ± 0.05 | 0.05 ± 0.05 |
| H+Al (cmolc/dm³) | 3.50 ± 0.66 | 2.58 ± 0.43 | 2.55 ± 0.45 | 2.88 ± 0.66 |
| M.O. (%) | 4.38 ± 0.64 | 4.18 ± 0.33 | 4.42 ± 0.28 | 4.33 ± 0.42 |
| SB (cmolc/dm³) | 6.61 ± 0.27 | 7.89 ± 0.72 | 7.06 ± 0.67 | 7.18 ± 0.77 |
| CTC(T) (cmolc/dm³) | 10.11 ± 0.58 | 10.46 ± 0.92 | 9.61 ± 1.06 | 10.06 ± 0.87 |
| V (%) | 65.55 ± 4.79 | 75.42 ± 3.20 | 73.56 ± 2.52 | 71.51 ± 5.55 |

*Values are mean ± SD. P and K by Mehlich-1 extraction; Ca, Mg, Al by KCl 1 mol/L; H+Al by SMP method; M.O. by loss-on-combustion; SB = sum of exchangeable bases; CTC(T) = cation exchange capacity at pH 7.0; V = base saturation.

**Supplementary Table S3. Sample sizes, descriptive statistics, and exact test statistics for alpha-diversity comparisons (Figures 4, 7, and 8).**

| **Figure** | **Season** | **Compartment / time** | **Index** | **n/grp** | **Maize mean±SD** | **Rotation mean±SD** | **Soybean mean±SD** | **KW p** | **Maize vs Rotation p** | **Maize vs Soybean p** | **Rotation vs Soybean p** |
| --- | --- | --- | --- | --- | --- | --- | --- | --- | --- | --- | --- |
| 4 | 3 (2018–2019) | Bulk soil | Chao1 | 4 | 2013±306 | 1769±202 | 2240±207 | 0.092 | 0.49 | 0.30 | 0.17 |
| 4 | 3 (2018–2019) | Bulk soil | Shannon | 4 | 7.11±0.137 | 6.99±0.103 | 7.19±0.105 | 0.118 | 0.49 | 0.49 | 0.17 |
| 4 | 3 (2018–2019) | Bulk soil | Pielou | 4 | 0.938±0.001 | 0.939±0.002 | 0.937±0.005 | 0.794 | 1.00 | 1.00 | 1.00 |
| 7 | 4 (2019–2020) | Rhizosphere, blooming | Chao1 | 12 | 1121±323 | 1087±259 | 1328±228 | 0.081 | 0.713 | 0.215 | 0.073 |
| 7 | 4 (2019–2020) | Rhizosphere, blooming | Shannon | 12 | 5.82±0.430 | 5.80±0.412 | 6.14±0.218 | 0.046 | 0.551 | 0.103 | 0.061 |
| 7 | 4 (2019–2020) | Bulk soil, blooming | Chao1 | 12 | 1737±496 | 1953±411 | 2112±432 | 0.225 | 0.44 | 0.30 | 0.44 |
| 7 | 4 (2019–2020) | Bulk soil, blooming | Shannon | 12 | 6.94±0.257 | 7.06±0.179 | 7.15±0.167 | 0.109 | 0.41 | 0.18 | 0.21 |
| 8 | 4 (2019–2020) | Rhizosphere, harvest | Chao1 | 12 | 3964±497 | 4241±356 | 3990±282 | 0.129 | 0.24 | 1.00 | 0.13 |
| 8 | 4 (2019–2020) | Rhizosphere, harvest | Shannon | 12 | 7.62±0.131 | 7.71±0.075 | 7.66±0.064 | 0.079 | 0.089 | 0.887 | 0.089 |
| 8 | 4 (2019–2020) | Bulk soil, harvest | Chao1 | 12 | 4232±410 | 4078±350 | 4360±407 | 0.219 | 0.38 | 0.38 | 0.30 |
| 8 | 4 (2019–2020) | Bulk soil, harvest | Shannon | 12 | 7.71±0.077 | 7.67±0.090 | 7.75±0.074 | 0.087 | 0.20 | 0.20 | 0.16 |

*Boxplots in Figures 4, 7, and 8 display the median and interquartile range (IQR); values below are mean ± SD per treatment group (n per group as indicated), with Kruskal–Wallis (KW) and pairwise Wilcoxon rank-sum test p-values (Benjamini–Hochberg adjusted). For Figures 2 and 3, error bars represent the standard error of the mean (SE); n = 4 blocks per treatment per season. Only one comparison reached conventional significance (Kruskal–Wallis p < 0.05): rhizosphere Shannon diversity at blooming (Figure 7), driven primarily by the soybean vs. rotation pairwise contrast (p = 0.061, marginal after BH adjustment).

**Table S4** Quality parameters for quantification of *Fusarium* spp. *fum1* (qPCR) in soil and maize stalks for all seasons.

| **Run number** | **Compartment** | **Threshold** | **R^2^** | **Efficiency** |
| --- | --- | --- | --- | --- |
| 1 | Maize stalk | 0.29 | 0.97 | 1.17 |
| 2 |  | 0.28 | 0.99 | 1.20 |
| 3 |  | 0.20 | 0.98 | 1.05 |
| 4 |  | 0.28 | 0.98 | 1.22 |
| 5 |  | 0.32 | 0.98 | 1.19 |
| 6 |  | 0.29 | 0.99 | 0.96 |
| 7 |  | 0.20 | 0.99 | 0.98 |
| 8 |  | 0.27 | 0.99 | 1.03 |
| 9 |  | 0.30 | 0.99 | 0.91 |
| 10 |  | 0.37 | 0.99 | 0.92 |
| 11 |  | 0.27 | 0.99 | 1.01 |
| 12 | Bulk soil | 0.28 | 0.99 | 1.07 |
| 13 |  | 0.22 | 0.99 | 1.03 |
| 14 |  | 0.15 | 0.99 | 0.97 |
| 15 |  | 0.19 | 0.99 | 1.04 |
| 16 |  | 0.17 | 0.99 | 0.96 |
| 17 |  | 0.21 | 0.96 | 1.17 |
| 18 |  | 0.19 | 0.96 | 1.17 |
| 19 |  | 0.19 | 0.99 | 0.95 |
| 20 |  | 0.16 | 0.99 | 0.79 |
| 21 |  | 0.14 | 0.99 | 0.99 |

*Runs performed using rotor gene 6000 thermocycler.

**Table S5** Summary of the two Illumina MiSeq sequencing runs and read processing.

| **Run** | **Samples included** | **n** | **FIGARO truncation F/R (nt)** | **Merged amplicon length** | **Library size after filtering (reads)** | **Normalized depth (SRS Cmin)** |
| --- | --- | --- | --- | --- | --- | --- |
| Run 1 | Seasons 1–3, bulk soil at harvest; season 4, bulk soil and rhizosphere at tasselling | 108 | 244/233 | about 430 bp | 13,096-47,233 (all samples) | 13,096 reads |
| Run 2 | Season 4, bulk soil and rhizosphere at harvest | 72 | 250/217 | about 430 bp | 13,096-47,233 (all samples) | 13,096 reads |

*Both runs: same DNA extraction kit, primers (Bakt_341F/Bakt_805R), library protocol, provider (Psomagen) and platform (Illumina MiSeq, 2 × 300 bp, V3 chemistry), with a target depth of about 20,000 reads per sample. Each run was denoised separately with DADA2; about 79% of merged reads were retained after denoising. No sample was excluded (n = 180). Raw reads for every sample, from which per-sample read counts can be obtained, are available in the NCBI Sequence Read Archive under BioProject PRJNA1122864.

**Table S6** Parameters used for processing of 16S rRNA amplicon sequences.

| **Step** | **Software (version)** | **Parameters** |
| --- | --- | --- |
| Primer removal | Cutadapt v2.8 | Primers Bakt_341F (5′-CCTACGGGNGGCWGCAG-3′) and Bakt_805R (5′-GACTACHVGGGTATCTAATCC-3′); maximum error rate 0.1 (default); minimum overlap 3 nt (default); untrimmed reads not discarded (default) |
| Read truncation | FIGARO (Weinstein et al. 2019) | Truncation positions chosen per run to maximize read retention while keeping sufficient overlap: run 1, forward 244 nt and reverse 233 nt; run 2, forward 250 nt and reverse 217 nt |
| Read merging | USEARCH v11.0.667, fastq_mergepairs | fastq_maxdiffs 5; fastq_pctid 90%; fastq_minovlen 16 nt; fastq_minlen 64 nt (defaults); no limits on merged length; merged amplicons about 430 bp |
| Quality filtering and denoising | QIIME2 v2020.2, q2-dada2 plugin (DADA2; Callahan et al. 2016), denoise-single on merged reads | Maximum expected errors (maxEE) 2.0; truncQ 2 (q2-dada2 defaults); error model learned separately for each sequencing run; singletons removed |
| Chimera removal | DADA2 (Callahan et al. 2016), removeBimeraDenovo | Method: consensus; minimum fold-parent-over-abundance 1.0 (q2-dada2 defaults) |
| Taxonomic classification | RDP classifier v2.13 (trainset 18) | Confidence threshold 0.8 (default) |
| ASV filtering | phyloseq / R | Removal of non-bacterial ASVs (chloroplast, mitochondria and ASVs not classified as Bacteria) |
| Normalization | SRS (Beule and Karlovsky 2020) | Cmin = 13,096 reads per sample (smallest library size); alternative: random rarefying to 13,096 reads (seed 913) |
| Read statistics | — | Run summary in Table S5; about 79% of merged reads were retained after DADA2; raw reads per sample in NCBI SRA (PRJNA1122864) |

*Both sequencing runs were processed with the same pipeline and parameters, except for the run-specific truncation positions. Analysis scripts are available at https://github.com/rafaelaguimaraes2/maize-soybean-rotation-microbiome.
